# Recall Accuracy in Children: Age vs. Conceptual Thinking

**DOI:** 10.3389/fpsyg.2021.686904

**Published:** 2021-08-10

**Authors:** Valeri Murnikov, Kristjan Kask

**Affiliations:** Laboratory of Experimental Psychology, School of Natural Sciences and Health, Tallinn University, Tallinn, Estonia

**Keywords:** child development, question types, conceptual thinking, investigative interviewing, eyewitness testimony

## Abstract

The aim of this study was to replicate a previous experiment using a different stimulus event. The present study examined the relationship between age, development of conceptual thinking, and responses to free recall, suggestive and specific option-posing questions in children and adults. Sixty-three children (aged 7–14) and 30 adults took part in an experiment in which they first participated in a live staged event, then, a week later, were interviewed about the event and tested using the Word Meaning Structure Test. Age and level of conceptual thinking were positively correlated in children. Compared to age, conceptual thinking ability better predicted children's accurate free recall and inaccurate responses to specific option-posing questions, but not inaccurate responses to suggestive questions.

## Introduction

Investigative interviews involve systematically gathering detailed, accurate accounts of a situation or event (Powell et al., 2005; St-Yves, 2014). It is known that children are more susceptible to suggestive questioning tactics than adults (Ceci and Friedman, 2000; Melnyk et al., 2007). A recent study (Kask et al., 2019) found a relationship between age, development of word meaning structure, and accurate detailed answers to free recall (prompting the child to tell with his/her own words such as “Tell me what happened”), suggestive (indicating a detail the child has not answered such as “The man was wearing a suit, didn't he?”) and specific option-posing questions (indicating a clearly inaccurate answer such as “Did he touch you?”).

Word meaning structure refers to the idea that the relationship between words and thoughts depends on the meaning of the word for the individual (Vygotsky, 1934; Luria, 1979). Two main types of concepts can be distinguished: everyday concepts and logical concepts (Vygotsky, 1934; see also Toomela, 2016a, 2017). With everyday concepts, word meaning is tied to sensory attributions of objects and actions observed in everyday situations. Logical concepts are more abstract, organized both hierarchically and logically. For example, if asking for the similarity between a cat and a dog, an answer in everyday concepts rely on sensory attributes (e.g., they go together because they have both four legs) whereas an example of the scientific concept would assume hierarchical relationship (e.g., they are both domestic animals). Through the development and specifically the education, the amount of logical concepts determine dominative conceptual thinking i.e., a person thinks mainly in everyday concepts or thoughts are mediated mostly by logical concepts.

The researchers demonstrated that children with dominative everyday conceptual thinking were less accurate in their responses to these questions. Both age and development of conceptual thinking were positively associated with answer accuracy. The aim of the present study was to replicate this prior experiment using a different stimulus event and an additional approach to measure the quality of witnesses' accounts.

Age and development of conceptual thinking (as measured by the Word Meaning Structure Test; Toomela, 2003) are closely related in children (Kask et al., 2019). The emergence of logical concepts qualitatively changes cognitive processes by allowing a child to mentally distinguish perceived information, to think independently about elements of perceived information, and to analyze abstract elements separately from perceived meaning (see Toomela, 2016a; Toomela et al., 2020). Those qualitative changes are important assumption for children to provide accurate accounts.

For children, providing an accurate account of a witnessed event is cognitively demanding (see Lindsay and Johnson, 1987), requiring efficient cooperation of many cognitive abilities, such as the ability to perceive and comprehend the initial event, to retain representations of the initial perception, to understand questions asked about the event, and to retrieve relevant information. Answering suggestive and specific option-posing questions requires discrimination between memories that were actually perceived and those that are based on imagination (see Lindsay and Johnson, 1987).

Reality monitoring is the process of discriminating memories from perceived vs. imagined events based on information quality (Johnson and Raye, 1981). This discrimination compares four information categories: (1) sensory information (properties and quantities of objects and subjects); (2) temporal information (the chronology of an event); (3) spatial information (object locations, subject locations, and their spatial relation); and (4) attributions (cognitive operations such as reasoning and thoughts about the event being described). According to the reality monitoring approach, accuracy of the memories depend on whether memories are based on information from the first three categories (i.e., perceived information or external sources) or the last category (i.e., imagination or internal sources).

Lindsay and Johnson (1987) found that the ability to provide accurate answers to free recall and suggestive questions is related to age, overall cognitive development, and the ability to mentally distinguish different sources of information. To date, studies evaluated the empirical evidence that the ability to provide accurate and detailed information is age-dependent (see e.g., Lamb et al., 2003, 2018). Results of a study by Kask et al. (2019) supported the notion that reality monitoring is age dependent and related to conceptual thinking.

Features of logical thinking correspond with cognitive abilities said to be crucial for accurate eyewitness accounts (Lindsay and Johnson, 1987); namely, perceiving and comprehending the event, retaining representations of the perception, retrieving relevant information, and discriminating accurate details. It has been shown that more dominant logical word meaning structure is related to better visual-spatial abilities (Tammik and Toomela, 2013; Toomela et al., 2020).

### Aim of the Current Study

In Kask et al. (2019), child participants watched a 30-s video and, 1 week later, were asked to recall what happened in the video clip and to answer suggestive and specific option-posing questions about the event. However, observing an event from a video clip is passive, whereas witnessing or falling victim to a real crime is active (even when the witness or victim does not realize the crime is happening). Thus, in our replication of the experiment, we chose to actively involve participants in the stimulus event; i.e., participants took part in a staged live event (see also Pompedda et al., 2021). In addition, we involved an adult sample to act as a control group.

The four main hypotheses were as follows: (H1) adults will recall more accurate and less inaccurate information in response to free recall, suggestive, and specific option-posing questions; (H2) Word Meaning Structure Test scores in children will be associated with age; (H3) Compared to age, Word Meaning Structure Test scores will better predict accurate answers provided by children in free recall and inaccurate answers to suggestive and specific option-posing questions; (H4) Word Meaning Structure Test scores will predict accurate answers provided by adults in free recall and inaccurate answers to suggestive and option-posing questions. The current study expands our understanding of conceptual thinking and how it influences the quality of children's accounts in investigative settings.

## Method

### Sample

Sixty three children (27 boys, 36 girls; mean age 10.35 years, *SD* = 2.24, range 7–14) and 30 adults (17 males, 13 females; mean age 25.93 years, *SD* = 7.54, range 19–45) participated in the study. Nineteen children were from Grade 1 (10 females, M = 7.47, SD =0.51, range 7–8); 26 were from Grade 3 (14 females, M = 10.54, SD = 0.51, range 10–11); and 18 were from Grade 7 (12 females, M = 13.11, SD = 0.47, range 12–14). Among the adults, 18 had finished secondary education, two had finished vocational education, and 10 had finished higher education. All participants' native language was Estonian, and they were recruited from Estonia's capital city, Tallinn, and its suburbs. The experiment was conducted in accordance with the Declaration of Helsinki and approved by the Tallinn Ethics Committee of Medical Research. Written consent was obtained from adult participants, parents of all children, and children over 10 years of age. Oral consent was obtained from children younger than 10 years of age.

### Materials

#### Stimulus Event

Participants took part in a science demonstration based on Jean Piaget's conservation of quantities of liquid test (Piaget, 1954). Before the experiment began, adult participants were guided to a room in Tallinn University, while children from participating schools took part in their home classroom. The stimulus event took place in two different locations at the university and in six different schools (see Appendix A for a detailed description of the stimulus event).

#### Interview

Participants were interviewed 1 week after the stimulus event by one of the five interviewers (three male, two female). All interviewers had participated in a training for interviewing purposes. After building rapport, the rules of the interview were introduced similarly to the NICHD protocol (Lamb et al., 2018). First, an invitation was offered (“*Some time ago, you saw a person who conducted a psychological demonstration. Tell me everything you remember about that event.”*). After the initial invitation, three more invitations were offered (“*Tell me more about it.”'/“What else do you remember about it?”*). To ensure the children were remembering the correct event (the school curriculum included several events each week), the invitation contained some information about the experiment.

Next, more specific questions were asked. We differentiated between suggestive (see Korkman et al., 2008) and specific option-posing questions (see Hughes-Scholes and Powell, 2008). Twelve suggestive and 12 specific option-posing questions were asked. Suggestive questions were questions in which the interviewer strongly communicated what response was expected, or questions in which the interviewer assumed answers that had not been revealed by the child (“*He was wearing a red shirt, wasn't he?”*). Specific option-posing questions focused the child's attention on incident-related issues but were misleading (e.g., containing inaccurate information; “*Did someone stand with him by the desk”*). The interview ended by discussing a neutral topic.

In free recall, answers were analyzed based on Johnson and Raye's (1981) reality monitoring approach, which distinguishes four types of information: sensory, spatial, chronological and attributions. Information from first three categories was categorized as accurate information. Coding was conducted as follows. Sensory information included details about a item's or person's properties and/or quantity. For example, the answer “*He took glasses* out of the *bag*” was coded as three details as it mentions persons action and two additional objects. Temporal information included sequential details about the story. For example, “He put the liquid in a longer glass *and then* he asked if they were equal now,” was coded as one detail. Spatial information included details about location of objects and subjects. For example, “A man came in *from the ceramics* room with one bag” was coded as one spatial detail.

To ensure interrater agreement, two independent coders each coded 60% of the overall responses of the answers to free-recall questions (i.e., 20% of the responses were coded twice) (Kendall's tau τ*b* = 0.824, *p* < 0.001).

Next, participants' answers to suggestive and specific option-posing questions (see Appendix B) were coded as either accurate or inaccurate (if the participant did not answer or did not remember/know then it was not considered inaccurate but removed from the analysis). The responses to suggestive and specific option-posing questions were coded by two independent coders who each coded 60% of the overall responses (i.e., 20% of the responses were coded twice) (Cohen's κ = 0.945, *p* < 0.001).

#### Word Meaning Structure Test

Word Meaning Structure Test measures development of conceptual thinking (Luria, 1979; Toomela, 2003; for a detailed description, see Kask et al., 2019). The test consists of three parts, with each part including six tasks. In the first part, participants were asked to explain the meaning of some concrete concepts (e.g., “school”) and some abstract concepts (e.g., “republic”). In the second part, participants were asked to evaluate how two concepts are similar. Some concepts were from the same category (e.g., “hammer—axe”) while others were complementary (e.g., “hat—head”). In the third part, participants were asked to choose two out of three words that belong together and explain their choices.

Responses were coded as everyday concepts if the explanation involved perceptual similarity, function, everyday activities, common relationships observable in everyday life, or if there was no answer. Responses were coded as logical concepts if the explanation involved a hierarchical relationship between words or associated the word with a hierarchically higher concept. Everyday concepts were coded as zero, and logical concepts were coded as one. A higher score reflected superior conceptual thinking. The maximum score was 18. Internal consistency was previously found to be acceptable, Cronbach's alpha = 0.77–0.86 (Toomela, 2003; Kask et al., 2019).

All responses were coded by two independent coders who each coded 60% of the overall responses (i.e., 20% of the responses were coded twice). Interrater agreement was acceptable (Cohen's κ = 0.909, *p* < 0.001), and internal consistency was good, Cronbach's alpha = 0.825.

#### Procedure and Coding

First, participants were informed of the following: the aims of the study; that data would be analyzed anonymously; that no personal information would be shared; that participation was voluntary; and that participation could be canceled at any time. Then, participants took part in the stimulus event which lasted about 10 min.

After 1 week had elapsed, participants were interviewed individually about the event in a separate room at the university or school. After the interview, the Word Meaning Structure Test was administered verbally. Participants were asked for their consent to video record the interview and the Word Meaning Structure Test. Two children refused to be video recorded but agreed to be audio recorded. The interview and test took about 15–20 min in total.

Finally, participants were thanked for their time and participation. The video and audio recordings were then transcribed and coded.

Four interviewers assisted data collection, all of them were blind to the aim and hypotheses of the paper. In total four coders who were also blind to the aim and hypotheses of the study coded the Word Meaning Structure Test (coded first, by two coders) and interviews (coded second, by another two coders). The data were presented to the coders without demographic information (age, gender, and education).

### Statistical Analyses

Data analysis was conducted using SPSS Version 27 by IBM. To test our hypotheses, we used the following analyses: Pearson correlation analysis; Kruskal-Wallis tests and Dunn *post-hoc* tests with Bonferroni correction; hierarchical linear regression analysis; and Cohen's *f*^2^ (Cohen, 1988). We use term “age group” if the variable was used categorically and “age” when continuously.

## Results

First, we used the Kruskal-Wallis test to check if responses to free recall, suggestive, and specific option-posing questions differed according to demonstrator, interviewer, or location. Results showed no effect of interviewers on responses (*H* < 5.77, *p* > 0.05). In adults, there was also no effect of location or demonstrator on responses (*H* < 0.894, *p* > 0.05). However, in children, there were effects of demonstrator [*H*_(1)_ = 4.46, *p* < 0.05] (Demonstrator 1, *M* = 8.21, *SD* = 1.61, *Mdn* = 9; Demonstrator 2, *M* = 7.36, *SD* = 1.56, *Mdn* = 7) and location [*H*_(5)_ = 14.55, *p* < 0.05] on the recall of accurate information for specific option-posing questions. To analyze the differences between six different locations, Dunn's pairwise tests were carried out (adjusting *p*-values using Bonferroni correction); results demonstrated that only Location 1 (*M* = 8.31, *SD* = 1.40, *Mdn* = 9) differed significantly from Location 2 (*M* = 6.63, *SD* = 1.54, *Mdn* = 7). These results indicate that, in the children's sample, results concerning accurate information for specific option-posing questions should be interpreted with care as more accurate information was recalled with a specific demonstrator and in a specific location.

### Free Recall

Next, age group differences in response to different questions were analyzed using Kruskal-Wallis tests (see Table 1). Statistically significant differences were found in accurate answers in response to free recall *H*_(3)_ = 21.51, *p* < 0.001 (*post-hoc* tests indicated that adults reported significantly more accurate answers than students in Grade 1).

**Table 1:** Children's and adults' responses to different question types.

**Category**	**Age 7**			**Age 10**			**Age 13**			**Adults**		
	**M**	**SD**	**Median**	**M**	**SD**	**Median**	**M**	**SD**	**Median**	**M**	**SD**	**Median**
Sensory	26.47a	14.46	24	41.04	17.94	42	44.22	25.68	34.5	68.83a	39.41	59.5
Temporal	2.47	1.87	2	2.96	1.95	3	3.39	3.33	2	3.57	4.42	3
Chronological	2.16a	1.61	2	4.20b	2.18	4.5	4.83c	3.35	4.5	5.33abc	3.95	5
Accurate free recall	31.10a	16.20	30	46.42	21.97	47	52.44	30.99	42.5	77.73a	45,26	72
Suggestive questions accurate answers	7.63	1.34	8	6.84	1.18	7	7.06	1.47	7	7.67	1.60	8
Suggestive questions inaccurate answers	3.58	1.22	3	3.68a	0.99	4	3.61b	1.42	4	2.42ab	1.48	2.5
Option-posing questions accurate answers	7.58	1.64	7	7.68	1.70	8	7.94	1.47	7.5	7.67	1.45	8
Option-posing questions inaccurate answers	3.21	1.08	3	2.56	1.42	2	2.67	1.46	2	2.37	1.22	2
Word Meaning Structure Test scores	1.00abc	1.33	1	3.96ad	2.71	3	5.67b	3.14	5	8.40cd	2.09	8

*a–d indicate statistically significant differences between age groups using Dunn's post-hoc tests with Bonferroni correction within each category (p < 0.05)*.

For children, both age and Word Meaning Structure Test scores were positively associated with accurate answers in response to free recall (*r* = 0.390, *p* < 0.01 and *r* = 0.517, *p* < 0.01, respectively). For adults, there was no association between age or Word Meaning Structure Test scores and accurate or inaccurate answers in response to free recall.

Next, the Kruskal-Wallis test was conducted to examine age group differences in different information categories. There was a significant difference in the amount of sensory information [*H*_(3)_ = 22.78, *p* < 0.001; *post-hoc* tests indicated that adults reported significantly more answers than students in Grade 1] and chronological information provided [*H*_(3)_ = 13.26, *p* < 0.01; *post-hoc* tests indicated that adults reported significantly more answers than students in all grades].

In children, age was positively correlated with the amount of sensory and chronological information provided (*r* = 0.398, *p* < 0.01; *r* = 0.435, *p* < 0.01, respectively). Word Meaning Structure Test scores were also positively correlated with sensory and chronological information (*r* = 0.515, *p* < 0.01; *r* = 0.419, *p* < 0.01). In adults, there were no correlations between age and Word Meaning Structure Test scores in any category of information.

### Age and Word Meaning Structure Test

Next, we analyzed associations between age groups, Word Meaning Structure Test scores, and number of answers in response to different question types. Word Meaning Structure Test scores varied as expected (*M* = 5.12, *SD* = 3.61, range 0–13), and internal consistency was good (Cronbach's alpha = 0.825). Word Meaning Structure Test (WMST) scores were as follows: for students in Grade 1 (*n* = 19, *M* = 1.00, *SD* = 1.33, range 0–5); for students in Grade 3 (*n* = 26, *M* = 3.96, *SD* = 2.71, range 0–9); for students in Grade 7 (*n* = 18, *M* = 5.67, *SD* = 3.14, range 0–12); and for adults (*n* = 30, *M* = 8.40, *SD* = 2.09, range 5–13).

According to the Kruskal-Wallis test, age groups differed significantly in Word Meaning Structure Test scores, *H*_(3)_ = 52.64, *p* < 0.001. *Post-hoc* tests indicated differences between: students in Grades 1 and 3 (*p* < 0.05); students in Grades 1 and 7 (*p* < 0.001); students in Grade 1 and adults (*p* < 0.001); and students in Grade 3 and adults (*p* < 0.001). Children's age and Word Meaning Structure Test scores were positively associated (Pearson), *r* = 0.599, *p* < 0.001; a similar association was not found in adults, *r* = 0.133, *p* > 0.484 (see Table 2).

**Table 2:** Pearson correlations between age, word meaning structure test scores, and responses to different question types in children and adults.

		**Age**	**Word Meaning Structure Test score**	**Free recall accurate answers**	**Sensory**	**Temporal**	**Chronological**	**Suggestive questions accurate answers**	**Suggestive questions inaccurate answers**	**Specific option-posing questions accurate answers**	**Specific option-posing questions inaccurate answers**
Children	Age		0.599^***^	0.390^**^	0.398^**^	0.209	0.435^**^	−0.186	0.019	0.031	−0.144
	Word meaning structure test score	0.599^***^		0.517^**^	0.515^**^	0.204	0.419^**^	−0.296^*^	−0.014	0.052	−0.318^*^
Adults	Age		0.133	0.183	0.231	−0.127	−0.063	−0.310	−0.115	0.058	−0.050
	Word meaning structure test score	0.133		0.117	0.142	0.016	−0.092	−0.031	−0.270	0.080	−0.005

a ***p < 0.001,

** *p < 0.01*,

* *p < 0.05*.

### Responses to Suggestive and Specific Option-Posing Questions

Finally, we analyzed age and Word Meaning Structure Test differences in response to different question types (see Table 1). The Kruskal-Wallis test indicated differences in inaccurate answers to suggestive questions [*H*_(3)_ = 13.79, *p* < 0.01; *post-hoc* test indicated that adults reported significantly fewer inaccurate answers than students in Grades 3 or 7].

Among children, Word Meaning Structure Test scores were negatively correlated with accurate answers in response to suggestive questions (*r* = −0.296, *p* < 0.05) and inaccurate answers in response to option-posing questions (*r* = −0.318, *p* < 0.05). For adults, there was no association between age and Word Meaning Structure Test scores to accurate and inaccurate answers in response to free recall, suggestive, or option-posing questions (*p* > 0.05).

To examine whether the number of inaccurate answers in response to suggestive or option-posing questions can be predicted according to age or Word Meaning Structure Test score, three hierarchical linear regression analyses were conducted (see Tables 3, 4).

**Table 3:** Predicting the number of inaccurate answers in children's answers in response to specific option-posing questions (*N* = 63).

	**Predictor**	***B***	***SE B***	**β**	***t***	***p***	***sr***
Step 1^a^	Age	−0.088	0.077	−0.144	−1.134	0.26	−0.144
Step 2^b^	Age	0.044	0.093	0.073	0.477	0.635	0.061
	Word Meaning Structure Test score	−0.159	0.067	−0.527	−2.367	0.021	−0.292

a *= R^2^ =.0 021, F_(1, 62)_ = 1.29, p > 0.05;*

b *= R^2^ = 0.104, F_(2, 62)_ = 3.49, p < 0.05, ΔR^2^ = 0.084, F_(1, 60)_ = 5.61, p < 0.05*.

**Table 4:** Predicting the number of accurate answers in children's answers in response to free recall (*N* = 63).

	**Predictor**	***B***	***SE B***	**β**	***t***	***p***	***sr***
Step 1^a^	Age	4.304	1.299	0.390	3.313	0.002	0.390
Step 2^b^	Age	1.388	1.511	0.126	1.194	0.362	0.118
	Word meaning structure test score	3.523	1.094	0.441	3.219	0.002	0.384

a *= R^2^ = 0.152, F_(1, 62)_ = 10.97, p < 0.01;*

b *= R^2^ = 0.277, F_(2, 62)_ = 11.51, p < 0.001, ΔR^2^ = 0.125, F_(1, 60)_ = 10.36, p < 0.01*.

The first hierarchical linear regression analysis tested if age and Word Meaning Structure Test scores predicted the number of inaccurate answers in response to suggestive questions for both children and adults. Age was entered at step one, and Word Meaning Structure Test score was entered at step two of the regression. At Step 1, the regression models for both children [*F*_(1, 61)_ = 0.021, *p* > 0.05, *R* = 0.019, *R*^*2*^ = 0.000] and adults [*F*_(1, 29)_ = 0.37, *p* > 0.05, *R* = 0.115, *R*^*2*^ = 0.013] were not statistically significant. Introducing Word Meaning Structure Test scores at Step 2 also resulted in non-significant models.

The second hierarchical linear regression analysis tested if age and Word Meaning Structure Test scores significantly predicted the number of inaccurate answers in response to specific option-posing questions for both children and adults. Age was entered at Step 1 and Word Meaning Structure Test score was entered at Step 2 of the regression. At Step 1, the regression models were not significant. Introducing Word Meaning Structure Test scores at Step 2 resulted in a significant model for children, *F*_(2, 62)_ = 3.49, *p* < 0.05, *R* = 0.323 (*R*^*2*^ = 0.104, Cohen's *f*^2^ = 0.116), where the partial correlation of inaccurate answers in response to specific option-posing questions with age was *r* = 0.07, *p* > *0.0*5, and with Word Meaning Structure Test scores was *r* = −0.537, *p* < 0.001 (see Table 3). In this model, only Word Meaning Structure Test scores were a statistically significant predictor (β = −0.361, *p* < 0.001).

Finally, as both age and Word Meaning Structure Test scores were significantly associated with accurate answers in free recall for children, the third hierarchical linear regression analysis tested if age and Word Meaning Structure Test scores significantly predicted the number of accurate answers in response to free recall. Age was entered at Step 1 and Word Meaning Structure Test scores were entered at Step 2 of the regression. At Step 1, the regression model was significant, *F*_(1, 62)_ = 10.97, *p* < 0.002, *R* = 0.390 (*R*^*2*^ = 0.152, Cohen's *f*^*2*^ = 0.179). Introducing Word Meaning Structure Test scores at Step 2 resulted in a significant model, *F*_(2, 62)_ = 11.51, *p* < 0.001, *R* = 0.527 (*R*^*2*^ = 0.277, Cohen's *f*^*2*^ = 0.294), where the partial correlation of inaccurate answers with age was *r* = 0.118, *p* > 0.05, and with Word Meaning Structure Test scores was *r* = 0.384, *p* < 0.01 (see Table 4). In this model, only Word Meaning Structure Test scores were a statistically significant predictor (β = 0.441, *p* < 0.01).

## Discussion

In this study, we aimed to replicate Kask et al.'s (2019) findings. First, we expected adults to recall more accurate and less inaccurate information in response to free recall, suggestive, and specific option-posing questions. We also hypothesized that, in children, Word Meaning Structure Test scores would be associated with age and would, compared to age, better predict children's responses in free recall, and to suggestive and specific option-posing questions. In adults, we expected Word Meaning Structure Test scores to predict responses to accurate answers in free recall and inaccurate answers to suggestive and open-posing questions.

The ability to think in logical concepts is connected to biological age, and development of concepts is further related to individuals' interaction with their sociocultural environment (for discussion, see Toomela, 2017). Our results confirm this notion. In children, age was positively correlated with Word Meaning Structure Test scores. According to Toomela (2016a), by the age of seven (Grade 1), the biological potential for the emergence of logical concepts occurs and increases simultaneously with the development of everyday concepts. By Grade 3, the biological potential to think in logical concepts is developed. Differences in logical conceptual thinking compared to older children and adults are related to the amount of logical concepts that have been internalized through interaction with the sociocultural environment and are specifically influenced by education. In our study, children in Grade 1 had fewer logical concepts available compared to children from other grades and adults; thus, results from our sample are consistent with this theoretical understanding.

Our results were consistent with findings that younger children are less detailed in their free narrative recall compared with older children (Lindsay and Johnson, 1987; Lamb et al., 2018; Kask et al., 2019). Only first-grade students' accuracy was significantly different from adults. Development of logical conceptual thinking was positively correlated with higher accurate number of answers to free recall, and Word Meaning Structure Test scores (but not age) were a significant predictor of higher accuracy in free recall. In adults, neither Word Meaning Structure Test scores nor age were related to accuracy.

These findings indicate that Word Meaning Structure Test scores can only predict quality of recalled information in some cases. At a certain developmental stage, a higher Word Meaning Structure Test score does not refer to the ability to think in logical concepts but rather refers to the amount of logical concepts available. In adults with higher education, Word Meaning Structure Test score variations could not predict the same phenomena as they could in children. Students in Grade 1 have only started to think in logical concepts, but for older children and adults, intragroup variability relies more on the proportion of logical and everyday concepts. Thus, adult cognition is mediated by logical concepts, and intermediate groups are comparable to adults in terms of their ability to think in logical concepts.

An important qualitative change in cognitive processes is the ability to think independently about elements of perceived information and to analyze them in abstract form, separately from perceived meaning (for discussion, see Toomela, 2016a; Toomela et al., 2020). This finding also addresses the importance of both biological development and interaction with environment. In our study, adults were mainly individuals with at least secondary or higher education; however, it could be expected that in adults with only primary education, features of thought might be similar primary school students (for a discussion about the impact of education on conceptual thinking, see e.g., Toomela et al., 2020). To address this feature of logical thinking, the relationship between reality monitoring categories and Word Meaning Structure Test scores should be further explored.

Our study found that sensory and chronological information were positively correlated with age and Word Meaning Structure Test scores. Adults provided more chronological information compared to children. Remembering an event's chronology may be more cognitively demanding than recalling other information since it requires mental organization of sensory and spatial information over time. Recalling sensory information assumes the ability to memorize information about objects, while recalling spatial information further demands memorization of an object's spatial relationship. Recalling chronological information requires the aforementioned sensory and spatial memorization, but adds a temporal dimension. This idea is consistent with the theoretical framework on conceptual thinking. For example, complex visuospatial abilities are highly related to the development of logical thinking (see Tammik and Toomela, 2013; Toomela et al., 2020).

Reality monitoring indicates that accuracy in answers to suggestive questions relies on the ability to discriminate between actual memories and imagined details. Conversely, inaccurate answers are often related to the fact that suggestive and specific option-posing questions tend to overwrite the perceived information (Lindsay and Johnson, 1987). Thus, the ability to provide accurate answers to suggestive questions relies on the ability to mentally discriminate perceived vs. imaginary information, and to analyze the elements of perceived information as different from the event being perceived. Therefore, in addition to age, Word Meaning Structure Test scores seem to be a theoretically suitable variable to consider when analyzing the accuracy of children's responses to suggestive and specific option-posing questions.

Similarly to Kask et al. (2019), our results showed that Word Meaning Structure Test scores were a strong predictor of inaccurate responses to specific option-posing questions, but unlike in Kask et al. not inaccurate responses to suggestive questions. We propose different explanations to this difference. First, it could be due to the nature of the stimulus event. In this study, the event was perceived live; compared to observing the stimulus event on video in Kask et al. (2019), observers in the current study had to organize more perceptual information from different sources. Second, a higher Word Meaning Structure Test score may indicate an observer's improved ability to discriminate between accurate and inaccurate information, which manifests in a better ability to inhibit inaccurate information (e.g., in response to specific option-posing questions). Third, children with higher Word Meaning Structure Test scores may be more aware of the social cues and therefore desire to respond an adult interviewer in an accurate way (bearing in mind that often in misinformation effect or suggestibility studies the children are interviewed repeatedly, Odegard and Toglia, 2013). Thus, this line of research should be examined further.

In sum, our findings are theoretically important for further research as age is often the primary or only variable in studies regarding developmental changes with regard to suggestibility in eyewitness testimony (e.g., Ceci and Bruck, 1993; Coxon and Valentine, 1997; Bruck and Ceci, 1999; Gudjonsson et al., 2016). Our findings showed that, compared to age, Word Meaning Structure Test scores are a better predictor of accuracy in recall and inaccurate answers to specific option-posing questions. Age is a coherent measurement that can predict the development of cognitive abilities; however, qualitative features of these abilities is key to understanding the nature of the relationship between age and information accuracy.

According to the concept of higher mental functions, cognitive abilities are semiotically mediated (a concept originally suggested by Vygotsky; for discussion, see Luria, 1969, 1979; Toomela, 2016a,b). Thus, qualitative features of cognitive abilities change as conceptual thinking is developed. Answering questions in investigative interviews relies on complex cooperation between different cognitive processes, thus the emergence of logical concepts and dominant logical word meaning structure is a crucial factor in the quality of recalled information.

## Data Availability Statement

The raw data supporting the conclusions of this article will be made available by the authors, without undue reservation.

## Ethics Statement

The studies involving human participants were reviewed and approved by Tallinn Ethics Committee of Medical Research. Written informed consent to participate in this study was provided by the participants' legal guardian/next of kin.

## Author Contributions

VM and KK designed the study, wrote, and critically reviewed the manuscript. VM created the materials and supervised execution of the experiment. KK analyzed and interpreted the data. Both authors contributed to the article and approved the submitted version.

## Conflict of Interest

The authors declare that the research was conducted in the absence of any commercial or financial relationships that could be construed as a potential conflict of interest.

## Publisher's Note

All claims expressed in this article are solely those of the authors and do not necessarily represent those of their affiliated organizations, or those of the publisher, the editors and the reviewers. Any product that may be evaluated in this article, or claim that may be made by its manufacturer, is not guaranteed or endorsed by the publisher.
