# Supplementary material for: Recall Accuracy in Children: Age vs. Conceptual Thinking
Source: Front Psychol. 2021 Aug 10;12:686904. doi: 10.3389/fpsyg.2021.686904 (PMC8382886; doi:10.3389/fpsyg.2021.686904)
Supplement: Supplementary file 1 [file Presentation_1.pdf]

Appendix A. *Detailed description of the stimulus event.*

One of two male science demonstrators (both in their early 20s, short light hair, similar height and weight, no facial hair, wearing a red t-shirt with a university logo and black jeans) entered the room with a black bag. The demonstrator introduced himself and asked whether the participants would be willing to participate in a science demonstration (*"Hello! My name is X and I study psychology at Tallinn University. I am here today to conduct a psychological demonstration. Do you agree to take part?"*).

From the bag, the man took out two glasses with the same width, one short and narrow glass, one bottle with red liquid, and one tray. He put them on a table while simultaneously describing his actions (*"Now I am taking two glasses out of the bag. /--/ Now I am taking the third glass out of the bag. /--/ Now I am taking a bottle with red liquid out of the bag /--/ Now I am taking a tray out of the bag"*). He then took the two glasses with his hands, walked from behind the desk closer to the audience, and asked from the audience whether the glasses were identical (*"First, I'll show you two glasses. Do they seem similar or different to you?"*). He started from the left side of the first row of seats, and ended on the right side of the first row of the seats.

After the audience responded, the demonstrator questioned *"Why do you think so?"* Then he took the bottle filled with red liquid and similarly showed the audience while asking, *"What is the color of this liquid?"* Then he filled both glasses with the red liquid and placed them on the tray while describing his actions (*"Now I'm pouring the liquid into this glass, and now into this glass").* Then he took the tray with two liquid-filled glasses on it, approached the audience, and asked whether the level of liquid was similar in both glasses (*"Please look at these glasses. Do you think this glass has a similar amount of liquid as this glass?").*

After the audience answered, he responded, *"Why do you think so?*" If participants said that the level is not equal, then the demonstrator corrected the level of liquid until the audience agreed that the level of liquid was equal across both glasses. Then, the demonstrator took a third glass which was narrower and taller, and emptied the contents of one wide glass into the tall narrow glass. Then he took the tray with one wide and one narrow glass on it, approached participants again, and asked which glass had more liquid (*"Please look at these glasses. Do you think this glass has a similar amount of liquid as this glass?").*

After the audience answered, he responded, *"Why do you think so?"* Depending on the answers, the demonstrator then briefly explained what this test demonstrates, asked the participants whether they had any questions, put all materials back in the bag, and left the room.

The sequence of the activities and the location of where the materials would be placed was pre-planned and rehearsed before the experiment started.

Appendix B. - *Suggestive and option-posing questions*

Suggestive questions are highlighted with the asterisk.

Did he speak fast?

*He had dark hair, didn't he? **

Was the liquid red?

He was wearing a red shirt, wasn't he?

*There were things on the table that weren't used in the experiment, right? **

Was anyone else standing next to the table with him?

*He showed the glasses close to everyone after pouring the liquid into the glasses, didn't he? **

Was the bottle with the red liquid on the left side of the table from your perspective?

*There was no one behind him, right? **

Did he have blonde hair?

*His bag was next to the table, wasn't it? **

Was he wearing a black shirt?

Did he take the bottle out of the bag first?

The glasses were at the beginning on the left side of the table from your perspective, weren't they?

*He placed glasses on the table at the beginning, didn't he? **

Did he show the glasses close to the back row first?

*That liquid was red, am I right? **

*He spoke slowly, didn't he? **

Did he have a high voice?

*He was quite young, wasn't he? **

Did he have long hair?

*He walked slowly in class, didn't he? **
